# Elevating crop disease resistance with cloned genes

**DOI:** 10.1098/rstb.2013.0087

**Published:** 2014-04-05

**Authors:** Jonathan D. G. Jones, Kamil Witek, Walter Verweij, Florian Jupe, David Cooke, Stephen Dorling, Laurence Tomlinson, Matthew Smoker, Sara Perkins, Simon Foster

**Affiliations:** 1 The Sainsbury Laboratory, Norwich Research Park, Colney Lane, Norwich NR4 7UH, UK; 2 The Genome Analysis Center, Norwich Research Park, Colney Lane, Norwich NR4 7UH, UK; 3 Cell and Molecular Sciences, The James Hutton Institute, Dundee DD2 5DH, UK; 4 School of Environmental Sciences, University of East Anglia, Norwich NR4 7TJ, UK

**Keywords:** potato, *Solanum*, GM, transgenic field trial, late blight resistance

## Abstract

Essentially all plant species exhibit heritable genetic variation for resistance to a variety of plant diseases caused by fungi, bacteria, oomycetes or viruses. Disease losses in crop monocultures are already significant, and would be greater but for applications of disease-controlling agrichemicals. For sustainable intensification of crop production, we argue that disease control should as far as possible be achieved using genetics rather than using costly recurrent chemical sprays. The latter imply CO_2_ emissions from diesel fuel and potential soil compaction from tractor journeys. Great progress has been made in the past 25 years in our understanding of the molecular basis of plant disease resistance mechanisms, and of how pathogens circumvent them. These insights can inform more sophisticated approaches to elevating disease resistance in crops that help us tip the evolutionary balance in favour of the crop and away from the pathogen. We illustrate this theme with an account of a genetically modified (GM) blight-resistant potato trial in Norwich, using the *Rpi-vnt1.1* gene isolated from a wild relative of potato, *Solanum venturii*, and introduced by GM methods into the potato variety Desiree.

## 1. Introduction

Of seven billion humans, too many—close to one billion—are hungry. The reasons are complex, and include poverty, poor governance, pre- and post-harvest losses and wastage, and climatic factors. Despite this complexity, food prices are influenced by supply and demand, and worldwide demand is dangerously close to exceeding available supply. As demonstrated by various reports in the past 5 years [1–3], although increasing supply will not be sufficient to address all the social factors that cause poverty, increased production is nevertheless required to avoid further increases in the number of hungry people. It has been estimated that we need to increase food production by 50% by 2030 and by 70–100% by 2050 [1–3]. Humans already appropriate approximately 25% of terrestrial photosynthesis for our own direct consumption, that of our domestic animals, and for forestry [4]. Given the long lead times for agriculture and scientific innovation to be converted into increased crop performance, investment in agricultural science and technology is urgent, and we need evidence-based and locally appropriate judgements about which tools and technologies should be deployed to meet the challenge. Different approaches to increasing crop production are appropriate in different locations. For example, in poor countries such as Malawi, fertilizers can greatly increase maize productivity [5], whereas in most developed countries, further increases in fertilizer application would be ineffective and wasteful. Along with many other challenges and options, we must sustainably reduce crop losses to weeds, pests and diseases. Without control, these factors reduce harvests approximately twofold [6].

Synthetic fungicides and insecticides substantially reduce crop losses. However, insecticides, in particular, can damage non-target organisms, including pollinators, and predators of pest insects. Plant breeders select for high-yielding crop varieties. With widespread use of agrochemicals to control crop disease, disease resistance usually receives less priority compared with optimizing the overall photosynthetic performance and harvest index of the crop. These priorities may need to change as increasingly stringent environmental legislation, particularly in Europe, is leading to a steady reduction in the repertoire of chemicals available for pest and disease control in crops. Disease pressure on potatoes and wheat in the UK is severe during wet summers such as that of 2012 (http://www.cropmonitor.co.uk). Potato farmers in northern Europe typically spray 10–15 (and up to 25) times per year to control late blight caused by the oomycete *Phytophthora infestans* (http://www.fera.defra.gov.uk/scienceResearch/scienceCapabilities/landUseSustainability/surveys/documents/arable2010.pdf) [7]. Application of agrochemicals imposes costs for chemicals, diesel fuel, machine maintenance and labour as well as causing soil compaction. In very wet conditions, it can be impossible to use tractors in the fields. Costs for late blight control have been estimated in the Netherlands to be around 330 Euros per hectare per year for chemicals alone [8], and will be similar in the UK. Plants harbouring late blight resistance genes may only require sprays for late blight towards the end of the season, which will reduce the associated costs and environmental impact substantially. However, spraying for other diseases such as early blight, and for weed control and fertilization, will usually or often still be required.

## 2. Host/pathogen coevolution

**Figure 1.**
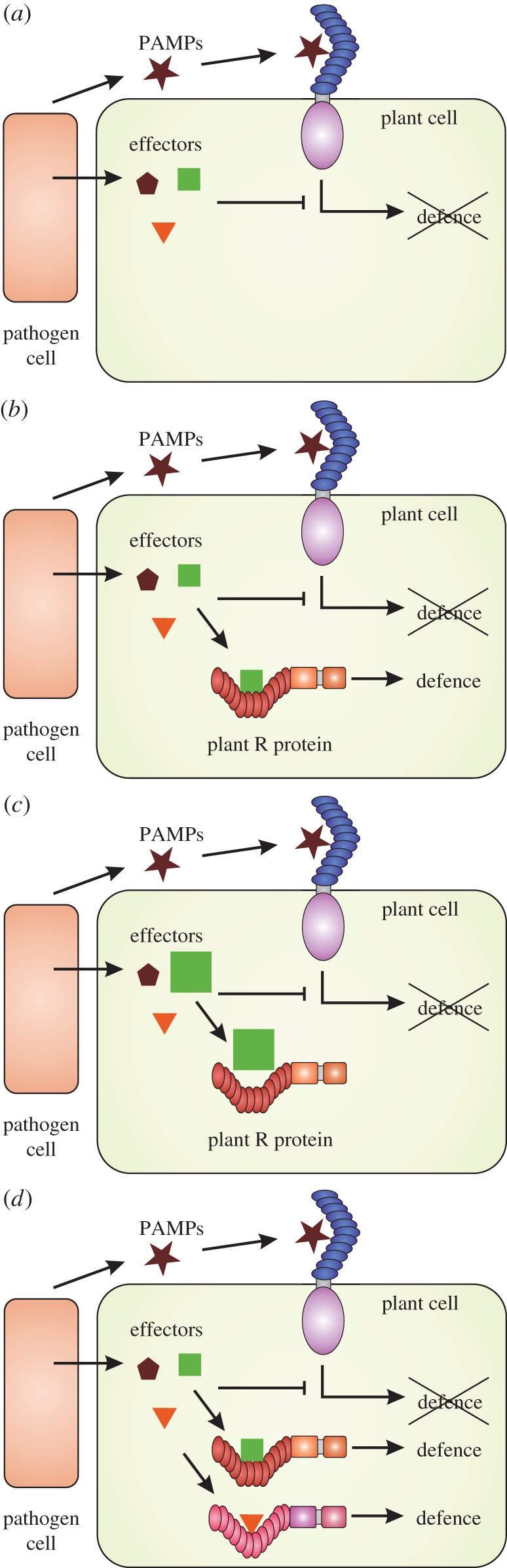
A simplified model of the role of plant *R* genes in plant–microbe interactions. (*a*) Pattern recognition receptors (PRRs) in the plant cell membranes confer recognition of pathogen associated molecular patterns (PAMPs), resulting in PRR-triggered immunity (PTI). Despite PTI, plants are susceptible to their pathogens owing to the delivery of effector molecules that attenuate this host resistance response. (*b*) Classical breeding for late blight resistance has focused on the introgression of single dominant *R* genes from wild sources such as *Solanum demissum*. These single genes resulted in strong selection on the pathogen effector genes, resulting (*c*) in the selection of mutated effectors that evade recognition, or in complete loss of recognized effectors. (*d*) In this paper, we postulate the cloning and transgenic stacking of several *R* genes. Stacked *R* genes will provide a more durable defence system, especially with several R gene stacks available to the breeder, because each *R* gene abolishes the selection for single effector mutations that circumvent a different *R* gene.

Plant/pathogen interactions exhibit three distinct phases [9,10] (figure 1). First, pathogens make relatively conserved molecules such as flagellin or chitin that plants have evolved the capacity to recognize. This recognition requires cell surface receptors that trigger defence upon recognition of these so-called pathogen (or microbe) associated molecular patterns (PAMPs or MAMPs). To succeed, pathogens must suppress host defence mechanisms, using molecules known as ‘effectors’ that are usually delivered into host cells but which can act in the intercellular spaces of the leaf (figure 1). Effectors interfere with defence processes directly, or with their activation, by disrupting host-signalling mechanisms. However (phase 3), plants have evolved the capacity to recognize specific effectors, either directly or indirectly by detecting their effects on host components [9]. This recognition is usually mediated by intracellular receptors encoded by disease resistance (*R*) genes. Pathogen effector complements and host *R* gene repertoires coevolve. For example, newly evolved *R* genes impose selection on recognized pathogen races for mutations in effectors that result in evasion of detection (figure 1). Pathogen races that evade detection by host immune receptors, in turn, select for hosts carrying new variant *R* genes that can detect either the new effector allele, or another effector. Hosts and pathogens have large *R* gene and effector repertoires, respectively. For example, the sequenced doubled-monoploid potato clone DM and its pathogen *Phytophthora infestans*, have more than 438 *R* genes and 563 effector candidates, respectively [11,12]. In nature, a high degree of genetic diversity (polymorphism) prevails in the host *R* gene and pathogen effector repertoires. Because of these polymorphisms, resulting in diversity of recognition capacity in the plant population, epidemics are rare for coevolving pathogens and hosts, because in any host plant population, most individual plants will be able to resist most of the circulating pathogen races. However, in agricultural ecosystems, populations of millions of genetically identical plants provide an ideal substrate for the growth of pathogen races that can evade recognition by the crop *R* gene repertoire.

Wild relatives of crop plants are a source of genes for disease resistance. The potato (*Solanum tuberosum*) can be crossed with a number of wild *Solanum* species; however, certain genetic restrictions apply. For example, the same ploidy level and genetic background must usually be present to generate viable crosses. The majority of pathogen resistances described to date were identified from wild *Solanum* species that are not directly crossable with the potato cultivar used in the field. Time-consuming and difficult bridge-crosses need to be made in this case, pointing to another problem caused by the heterozygous tetraploid nature of potato varieties [13]. This means that when a potato with a desired combination of characteristics is crossed to another, it is extremely difficult to recover the beneficial parental combination of alleles in the progeny. It has therefore usually proved difficult to breed in desired traits such as disease resistance without sacrificing good combinations of alleles at other loci. The potato cultivars Bionica and Toluca are very good examples for this breeding process, into which the durable late blight resistance gene *Rpi-blb2* has been introgressed from a diploid wild species *Solanum bulbocastanum*. However, this process took more than 30 years of crossing and selection to obtain the final product [13]. There is great value in genetic approaches that can improve disease resistance in good potato varieties without disrupting favourable combinations of alleles.

Although this article emphasizes classical disease resistance genes, the first layer of plant defence pattern recognition receptor-(PRR) triggered immunity (PTI) can also provide a useful source of elevated disease resistance. All plants have the capacity to activate defence upon recognition, via pattern recognition receptors (PRRs), of PAMPs/MAMPs such as fungal chitin or bacterial flagellin. However, not all plant families have the same repertoire of PRRs; most plants are unable to recognize the bacterial translation initiation molecule elongation factor-Tu (EF-Tu), but this recognition is found in the Brassicaceae, including the model species *Arabidopsis*. Recognition requires the PRR EF receptor (EFR), which encodes a transmembrane receptor kinase that exercises surveillance at the plant cell surface. When the *Arabidopsis EFR* gene is transgenically introduced into tomatoes, the resulting tomato lines show substantially enhanced resistance to bacterial diseases caused by *Ralstonia solanacearum* and *Xanthomonas* sp. [14]. This simple route to enhanced bacterial resistance could have broad utility.

Potato late blight resistance genes (resistance to *Phytophthora infestans* (*Rpi*) genes), confer recognition of specific *P. infestans* effectors, and use this recognition to trigger activation of defence, which arrests pathogen growth [15]. Mutations in the gene that encodes this effector can result in its elimination or alteration, enabling the pathogen to evade detection by the *Rpi* gene, with the result that the *R* gene is no longer effective at controlling races of the pathogen that lack the recognized form of this effector.

If it is so easy to mutate recognized effectors to evade *R* genes, then it is remarkable that any are retained by evolution. Furthermore, not all *R* genes are rapidly overcome; some are more ‘durable’. Why are some *R* genes more durable than others? This is almost certainly because some effectors are more indispensable to the pathogen than others. From analysis of the effector repertoire of pathogens, enabled by advances in DNA sequencing methods, we can often predict effectors from their protein sequence motifs. For example, *P. infestans* effectors have a signal peptide and a so-called RxLR amino acid sequence motif close to the signal peptide cleavage site in the secreted effector [15]. If a particular effector is shared between multiple races of a pathogen, then it is more likely to be indispensable for the pathogen than an effector that is present in some but not all races.

This knowledge provides a crucial tool for discovering the most indispensable pathogen effectors, and prioritizing *Rpi* genes that recognize these effectors for deployment in crops. The importance of the *P. infestans* Avr3a effector was directly examined by testing the virulence of races in which the *Avr3a* gene had been silenced. Remarkably, *P. infestans* lines with reduced levels of Avr3a expression are either weakly virulent or completely non-virulent [16]. Avr3a is found in two allelic forms [15]; Avr3a^KI^ and Avr3a^EM^ (varying in two amino acid positions). The *Rpi* gene *R3a* confers recognition of the Avr3a^KI^ form but not the Avr3a^EM^ form [15]. The Avr3a^EM^ form is present in the widespread virulent race 13_A2, which is why it can overcome *R3a* [16]. Efforts are underway in at least two laboratories to identify novel forms of *R3a* that can recognize both Avr3aKI and Avr3aEM forms, either by screening wild *Solanum* populations or from accelerated evolution in the laboratory followed by transient Agrobacterium-mediated assays of *R3a* and *Avr3a^EM^* alleles. In addition, *Rpi* genes exist that confer useful resistance against most known races of *P. infestans*, notably *Rpi-blb1, Rpi-blb2* and *Rpi-vnt1* [17–20]. Rare races exist that can overcome one or another of the *Rpi* genes, but no race exists that can overcome all three.

## 3. Advantages of genetically modified deployment of *Rpi* genes

Breeders have typically introduced one *Rpi* gene at a time from wild relatives into cultivated potato. Each introduction is laborious and slow, and so far has resulted in an *Rpi* gene that is overcome by new *P. infestans* races in less time than it took to breed the new variety that contains it. It is usually not known whether a resistance-breaking strain was present prior to deployment of the new *Rpi* gene, or whether new mutations arose and were selected by the *Rpi* gene once these lines were planted over a sufficiently large area. Understandably, breeder's enthusiasm for using single major *Rpi* genes is now low, because these genes are often not durable, and breeding multiple, independent new *Rpi* genes is even more difficult than breeding one at a time.

However, the prospects for using dominant major *Rpi* genes for disease control have been greatly improved by recent advances. Previously, breeders were unable to prioritize *Rpi* genes that recognized the pathogen's most indispensable effectors; now they can. Moreover, if one can combine multiple *Rpi* genes on one DNA construct (‘stacking’), then each gene can be expected to reduce the selection pressure against the other genes on the construct [21]. Furthermore, with genetically modified (GM) methods, one can insert the *Rpi* gene stack into a favoured variety and recover derivatives with all the properties of that favoured variety, but with the addition of blight resistance. By contrast, breeding not only breaks up favourable combinations of alleles in a variety of choice, but may introduce deleterious alleles of genes linked to the novel disease resistance, which are difficult to eliminate from subsequent breeding steps. If one knows the identity of each recognized effector, the function of each of the transgenic *Rpi* genes can be verified by transient delivery of the cognate effector and testing for activation of defence. This would not be possible merely by using *P. infestans* disease assays, because each *Rpi* gene is effectively epistatic to (masks) the other *Rpi* genes in the stack. Finally, in principle, it may be possible to deliver *Rpi* alleles that recognize all alleles of indispensable effectors such as Avr3a. In combination, these approaches provide a technology that should enable the creation of potato varieties that will be extremely difficult for *P. infestans* to evolve to colonize.

## 4. First steps: a genetically modified blight-resistant potato trial in Norfolk, UK

We isolated the *Rpi-vnt1.1* gene from *Solanum venturii* [19] and the *Rpi-mcq1* gene from *Solanum mochiquense* [22]. *Rpi-vnt1.1* is known to confer resistance to race 13_A2 and 6_A1 in detached leaf assays (DLAs). Transgenic potato var. Desiree plants carrying either *Rpi-mcq1* or *Rpi-vnt1.1* were produced during the course of verifying the isolation of these genes [19]. Plant and tuber phenotypic traits in *Rpi-vnt1.1*-transgenic lines were practically identical to the non-transformed Desiree. No differential plant characteristics (flower colour, leaf type, foliage colour) or tuber (shape, size, flesh colour, skin type) were noted between the transgenic line and the non-transformed controls. Similarly, no major differences in total yield were observed between transgenic and non-transgenic plants grown in pathogen-free areas (data not shown).

Although some Ecuadorian *P. infestans* strains exist that do not express the cognate effector *Avr-vnt1* and which can overcome resistance conferred by *Rpi-vnt1.1*, such races are not present in Europe. *Rpi-mcq1*, on the other hand, does not confer resistance to 13_A2 and 6_A1 in DLAs, but we still wished to test whether it conferred partial resistance in the field. After obtaining permission for a GM potato trial from the Department for Environment, Food and Rural Affairs under licence 10/R29/01, and building a securely fenced area in the John Innes Centre field plots, a field trial was undertaken during 2010, 2011 and 2012. Each year, 192 GM potato plants were planted, surrounded by non-transgenic potato plants of both Desiree and Maris Piper varieties. We did not inoculate with late blight, but waited for races circulating in the UK to blow in.

We considered the effect of temperature, total rainfall and humidity on the *P. infestans* infection pressure in the field experiments. The so-called Smith period defines the optimal conditions for late blight occurrence, sporulation and infection progress. A Smith period is observed when on two or more consecutive days, the minimum temperature is 10°C or above, and on each day the relative humidity is greater than 90% for at least 11 h (http://www.Blightwatch.co.uk). We noted strong correlation between weather conditions and results of our field trial experiment (for details of weather conditions see the electronic supplementary material, figure S1).

**Figure 2.**
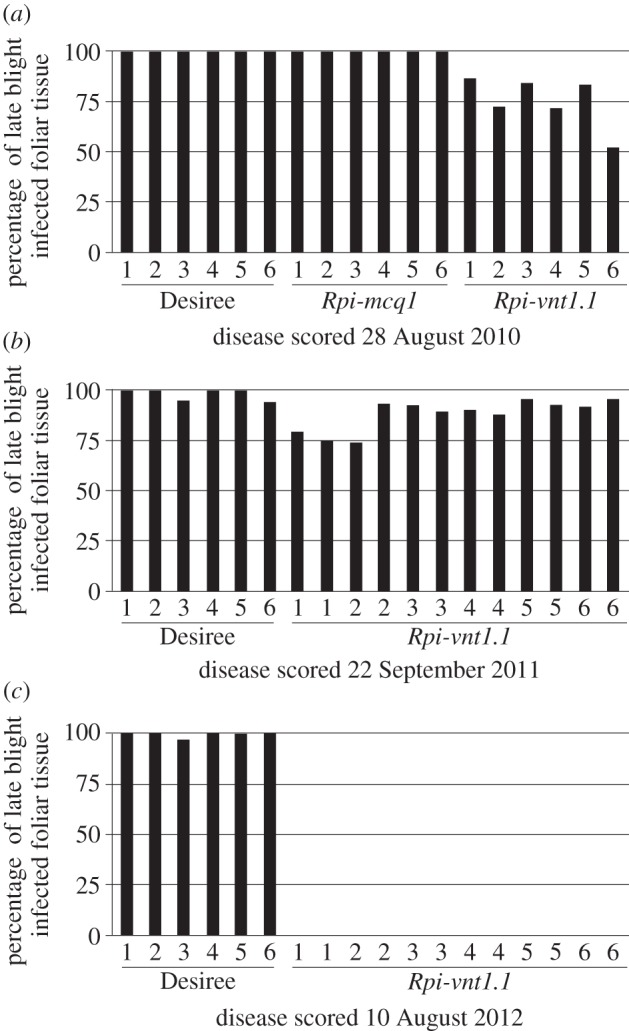
Comparison of disease severity in *Rpi-vnt1.1*-transgenic and non-transgenic Desiree plants. Data are average disease severity scores from 16 plants (single block) displayed as a percentage of necrotic tissue. The title of the *x*-axis represents the sampled plot number. All plants were scored as indicated in the figure. While blight arrived in (*a*) 2010 and (*c*) 2012 during the vegetative growth, it arrived at the end of this period in (*b*) 2011, when plants were already senescing.

**Figure 3.**
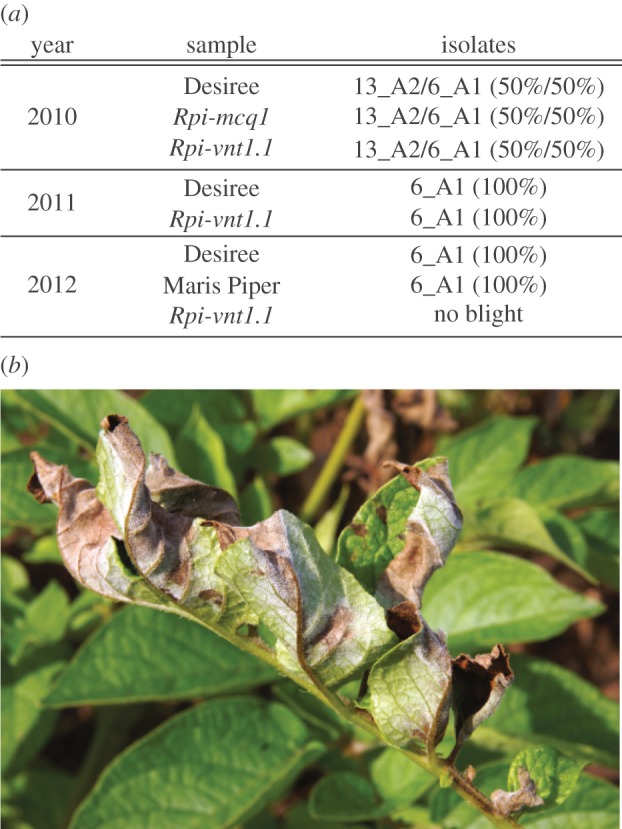
Survey of *Phytophthora infestans* strains isolated from transgenic *Rpi-vnt1.1*, *Rpi-mcq1* and non-transgenic Desiree and Maris Piper plants. *Phytophthora infestans* samples (*a*), 10 for each type of plants, randomly chosen from all plots, were collected from actively sporulating areas (*b*) into fast technology for analysis of nucleic acid cards.

During the first year of the experiment, we tested both *Rpi-vnt1.1* and *Rpi-mcq1*. Conditions were not ideal; the GM plants in the plots were all transplanted from pots in the glasshouse, rendering them physiologically different from non-GM Maris Piper and Desiree plants that sprouted from tubers. Weather conditions during that year were mostly unfavourable for late blight occurrence. June and most of July were warm and dry, with low relative humidity. While daily minimum temperatures remained above 10°C for nearly all of the extended period 6 June 2010 through to the end of August, average daily relative humidity never reached 90% over this same period (38–88%) and on only one day, 10 June, did relative humidity at midnight reach 90%. First symptoms of late blight were observed in mid-August, which correlated with higher rainfall and lower daytime maximum temperatures at the end of July and during the first weeks of August. Disease severity was scored after three weeks of favourable weather conditions for late blight. As shown in figure 2*a*, the *Rpi-vnt1.1* transgenic lines showed reduced disease severity, varying from 50% to 80% of plant tissue infected in different plots, in contrast with full susceptibility of the non-transformed Desiree line with 100% of infected tissue (dead plants). The *Rpi-mcq1* did not confer resistance in the field experiment, displaying 100% susceptibility, similar to non-transgenic control plants. This was not a surprising finding, as this gene did not confer resistance in laboratory experiments using DLAs against the two strains detected under field conditions (figure 3), 13_A2 and 6_A1. Therefore, we decided not to include *Rpi-mcq1* in subsequent field trials in 2011 and 2012, instead increasing the number of *Rpi-vnt1.1* transgenic plants to 2 × 16 blocks. Since late blight arrived in mid-August 2010, when plants were already full-grown, and tubers mostly developed, we did not observe any differences in tuber yield between transgenic *Rpi-vnt1.1*, transgenic *Rpi-mcq.1* and Desiree control (data not shown).

The weather conditions during the second year of the experiment (2011) were even less favourable for late blight (for details, see the electronic supplementary material, figure S1). Spring 2011 in East Anglia had been exceptionally dry (the driest on record http://www.metoffice.gov.uk/climate/uk/datasets/Rainfall/ranked/East_Anglia.txt) and July in particular was then a little cooler than average with a daily minimum temperature often less than the 10°C minimum required for a full Smith period. This resulted in late blight not being observed until the first week of September, when plants had already started senescing. In these circumstances, the effect of the transgene was moderate. *Rpi-vnt1.1* transgenic plants showed slightly elevated disease resistance on three blocks only, with 75% leaf area infected, in comparison with 95–100% infected tissue in non-transformed Desiree and the remaining nine blocks of *Rpi-vnt1.1* transgenic plants (figure 2*b*). During that year, only the 6_A1 race was detected in field conditions, whereas it composed 50% of blight strains detected in the previous year. In addition, DLAs showed that *Rpi-vnt1.1* gene confers resistance to that isolate. Therefore, high susceptibility of plants is owing to late occurrence of *P. infestans* at the beginning of plant senescence, rather than owing to infection with a virulent strain. As in the previous year, we did not see differences in tuber yield between transgenic and non-transgenic Desiree plants (data not shown).

**Figure 4.**
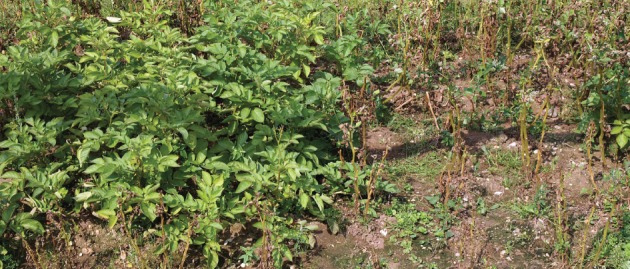
*Rpi-vnt1.1*-transgenic and non-transgenic Desiree in field trials. Photograph was taken on 10 August 2012, almost one month after first symptoms of infection on Desiree plants were observed (13 July 2012). No symptoms of late blight were observed on transgenic plants, neither when photographs were taken nor towards the end of the experiment. Left, transgenic plants; right, non-transgenic.

**Figure 5.**
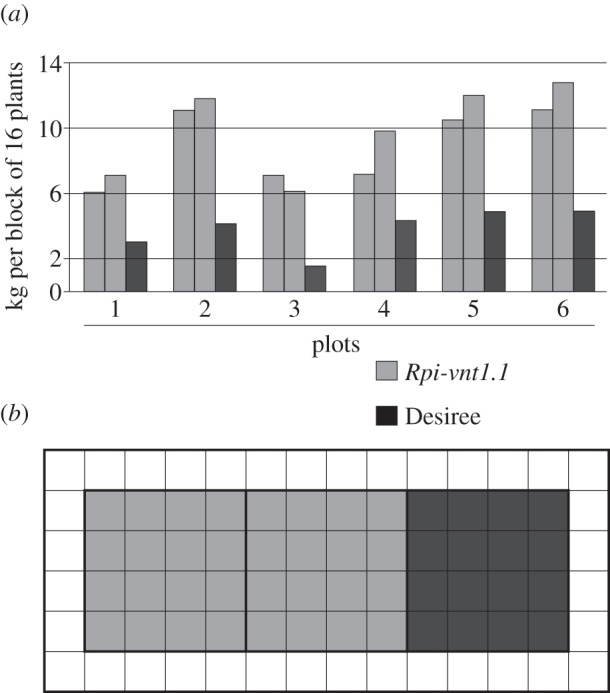
Comparison of yield in *Rpi-vnt1.1*-transgenic and non-transgenic Desiree plants. Total yield is in kilogram per block (16 plants, *a*). Each plot (*b*) consisted of two blocks of transgenic *Rpi-vnt1.1* (light grey) and one block of Desiree (dark grey), surrounded by one or two rows (external borders) of Maris Piper (white). Transgenic and non-transgenic blocks in each plot were planted in random order. Tubers from each block were collected and weighted separately.

The last year of the field trial, 2012, had the most advantageous weather conditions for *P. infestans* infection (see the electronic supplementary material, figure S1), with high rainfall (it was provisionally the second wettest July and fifth wettest summer in 100 years in the East Anglian region; http://www.metoffice.gov.uk/climate/uk/datasets/Rainfall/ranked/East_Anglia.txt) and high relative humidity two to three weeks before the first symptoms of late blight appeared (13 July). Additionally, late blight occurrence was preceded by five consecutive full Smith periods (6–11 July; http://www.Blightwatch.co.uk), conditions not observed in previous field trial years. Plants were scored for disease severity after nearly one month, on 10 August. During that time, an additional four full Smith periods were observed, but there was also one week of high maximum temperature (up to 25°C) without any rainfall (21–27 July). As shown in figure 2*c*, even with such strong infection pressure owing to perfect ‘blight weather’, *Rpi-vnt1.1* transgenic plants remained fully resistant to late blight. No signs of infection were observed either before scoring time or till the end of the experiment, when tubers were collected in the first week of October. Disease progressed quickly on non-transgenic Desiree plants, with 100% infected tissue observed on 10 August, when almost all plants were already dead and without any foliar tissue (figure 4). Early infection during the tuber growth phase also led to a severe drop in yield in non-transgenic plants. For *Rpi-vnt1.1* transgenic plants, the total weight of tubers from 16 plants varied from 6 to 13 kg, depending on the block, while it showed 50–75% reduction in the case of non-transgenic plants, yielding from 1.6 kg to a maximum of 5 kg in tuber weight (figure 5). As no infection was observed on *Rpi-vnt1.1* transgenic plants, only samples from Desiree and Maris Piper were collected for isolate determination. Again, the dominant isolate was genotype 6_A1, as in the second year of the trial.

These data show that Desiree potato lines transgenic for the *Rpi-vnt1.1* blight resistance gene from a wild relative of potato, *S. venturii*, can confer resistance in the field to races of late blight that circulate in the UK. The predominant race was 13_A2 in 2010, and 6_A1 in 2011/2012. The data do not prove that *Rpi-vnt1.1* alone will be sufficient to protect the UK or any other potato crop in perpetuity; indeed, this is highly unlikely. However, they do show that the *Rpi-vnt1.1* gene is functional in the field in three successive seasons in small plots. In addition, this proves that it is possible to transfer stably an *Rpi* gene from a wild relative into a cultivated potato variety without altering its functionality.

## 5. Discussion

The proposal to use GM methods to deploy disease resistance genes from wild relatives of crops is not new [8]. BASF developed a potato variety, Fortuna, that carries *Rpi-blb1* and *Rpi-blb2*, and showed excellent field resistance. In comparison with the previously described Bionica and Toluca [13], this cultivar not only harbours a second functional *R* gene, but was also created in much shorter time. Unfortunately, BASF have concluded that the obstacles and costs of bringing this blight-resistant Fortuna variety to market in Europe are too high to justify further investment in the project. This may be a good outcome for shareholders of agrichemical companies and tractor manufacturers, but not for farmers or for the reduction of the environmental impact of agriculture.

It is noteworthy how little blight was observed in 2011, and how even in a wet year like 2012, when farmers spray approximately 15 times, blight might not set in till mid-July. This illustrates two points. First, spraying, after a Smith period, can be difficult, and the need for spraying would be reduced with a fully blight-resistant variety. Second, in a dry year like 2011, blight can still appear at the end of the season, and once plants are senescing, because defence is an active process, the presence of an *R* gene does not guarantee resistance. Therefore, farmers might be advised to spray even resistant varieties towards the end of a season to ward off infections that might result in tuber blight and consequent storage losses.

Other examples exist of gene transfer from one species to another to confer useful disease resistance. The pepper *Bs2* gene for *Xanthomonas* resistance was isolated and shown to confer *Xanthomonas* resistance when transgenically introduced into tomatoes, a species that carries very limited natural genetic variation for resistance to this disease [23]. Broadly, this general approach of moving disease resistance genes from one plant species to another has great potential to benefit agriculture by replacing chemical control with genetic control.

How many crop/pathogen systems might be amenable to this kind of approach? Perhaps the greatest opportunity lies in cereal rusts. Wheat stem and striped rusts caused by *Puccinia graminis* and *Puccinia striiformis* pose substantial danger to the world's wheat supplies, particularly in less developed countries. Losses can be almost complete and epidemics explosive, owing to the vast number of spores produced during a successful infection. A new strain of stem rust (Ug99) emerged in Africa in 1999 and has spread to the Middle East but not (so far) to the Punjab. The arguments made earlier for GM approaches apply to this system. A substantial contribution to reducing losses could be obtained if multiple independent *R* genes were cloned, shown to recognize distinct and relatively immutable and indispensable pathogen effectors, and transformed in a stack into an elite variety. Several groups around the world are now collaborating to achieve this goal, starting with resistance genes to Ug99 defined in wild relatives of wheat from the Sitopsis section of the *Aegilops* genus, such as *Aegilops sharonensis* [24,25] (http://www.2blades.org). As genomics methods continue to be refined, and as knowledge of the genomes of crops and their relatives continues to expand, resistance gene isolation will get easier. These genomics methods will also facilitate characterization of the genomes of pathogens such as rusts and oomycetes, and in particular will enable a full understanding of the genetic diversity in these organisms and the differences in effector allele complements that underpin why one race can overcome an *R* gene that another cannot. From this information, and from devising good assays to test which effector candidate is recognized by which *R* gene, cassettes of *R* genes with the highest probability of durability can be stacked and deployed. The prospects for tipping the evolutionary balance in favour of the crop and against the pathogen are good.

## 6. Material and methods

### (a) Plant material

*Rpi-vnt1.1* transgenic potato plants were as described in [19]. The *Rpi-mcq1* resistance gene was isolated from *S. mochiquense* as described in [22], and plants of the potato cultivar Desiree were transformed with the binary vector pSLJ21153, carrying the full sequence of *Rpi-mcq1* including its native promoter and terminator sequences [19]. Transformants were selected as described in [19]. One selected line per each transgene was used in the field trial. Using PCR on gDNA and semi-quantitative RT-PCR on total RNA, we confirmed the absence of vector backbones and that transgenes are expressed on a very low level (data not shown). Each year, gDNA was extracted from sprouts of 10 randomly chosen tubers and tested for presence of the transgene [19].

Potato cultivar Maris Piper was planted as guard crop. All tubers were kept at 6**°**C between experiments and left in a glasshouse one week prior planting.

### (b) Field layout

Tubers were planted in plots consisting of three blocks, two transgenic and one non-transgenic control, with each 16 plants. One row of the guard crop Maris Piper was planted between plots and two rows were surrounding all plots. Plants were spaced with 40 cm within and between rows. The block organization within the plots was random and the plot changed every year. See figure 5*b* for details.

### (c) *Phytophthora infestans* isolates and detached leaf assays

Isolates of the multi-locus genotypes 13_A2 and 6_A1 were kindly provided by Cooke [16]. DLAs on transgenic plants were carried out as described in [26].

### (d) Field survey of *Phytophthora infestans* strains

*Phytophthora* samples were randomly chosen from all plots and collected from actively sporulating areas into fast technology for analysis of nucleic acid cards. Analysis was done according to [27].

### (e) Assessment of disease severity in field conditions

Disease severity was determined as described for whole plant assays in [28].

### (f) Weather data

Daily maximum and minimum 2 m air temperature, daily rainfall and hourly relative humidity data were taken from an agro-meteorological weather station located 15 km northeast of the field trial site, in an agricultural setting away from the coast, over the period May–September 2010–2012. Long term monthly weather records for the East Anglia region, maintained by the UK Meteorological Office and covering the period since 1910, were also consulted for context. The latter are provisional from summer 2012 onwards.
